# Elucidating the Ticking of an In Vitro Circadian Clockwork 

**DOI:** 10.1371/journal.pbio.0050093

**Published:** 2007-03-27

**Authors:** Tetsuya Mori, Dewight R Williams, Mark O Byrne, Ximing Qin, Martin Egli, Hassane S Mchaourab, Phoebe L Stewart, Carl Hirschie Johnson

**Affiliations:** 1 Department of Biological Sciences, Vanderbilt University, Nashville, Tennessee, United States of America; 2 Department of Molecular Physiology and Biophysics, Vanderbilt University, Nashville, Tennessee, United States of America; 3 Department of Pharmacology, Vanderbilt University, Nashville, Tennessee, United States of America; 4 Department of Biochemistry, Vanderbilt University, Nashville, Tennessee, United States of America; University of Geneva, Switzerland

## Abstract

A biochemical oscillator can be reconstituted in vitro with three purified proteins, that displays the salient properties of circadian (daily) rhythms, including self-sustained 24-h periodicity that is temperature compensated. We analyze the biochemical basis of this oscillator by quantifying the time-dependent interactions of the three proteins (KaiA, KaiB, and KaiC) by electron microscopy and native gel electrophoresis to elucidate the timing of the formation of complexes among the Kai proteins. The data are used to derive a dynamic model for the in vitro oscillator that accurately reproduces the rhythms of KaiABC complexes and of KaiC phosphorylation, and is consistent with biophysical observations of individual Kai protein interactions. We use fluorescence resonance energy transfer (FRET) to confirm that monomer exchange among KaiC hexamers occurs. The model demonstrates that the function of this monomer exchange may be to maintain synchrony among the KaiC hexamers in the reaction, thereby sustaining a high-amplitude oscillation. Finally, we apply the first perturbation analyses of an in vitro oscillator by using temperature pulses to reset the phase of the KaiABC oscillator, thereby testing the resetting characteristics of this unique circadian oscillator. This study analyzes a circadian clockwork to an unprecedented level of molecular detail.

## Author Summary

Circadian biological clocks are present in a diverse range of organisms, from bacteria to humans. A central function of circadian clocks is controlling the adaptive response to the daily cycle of light and darkness. As such, altering the clock (e.g., by jet lag or shiftwork) affects mental and physical health in humans. It has generally been thought that the underlying molecular mechanism of circadian oscillations is an autoregulatory transcriptional/translational feedback loop. However, in cyanobacteria, only three purified clock proteins can reconstitute a circadian rhythm of protein phosphorylation in a test tube (in vitro). Using this in vitro system we found that the three proteins interact to form complexes of different compositions throughout the cycle. We derived a dynamic model for the in vitro oscillator that accurately reproduces the rhythms of complexes and of protein phosphorylation. One of the proteins undergoes phase-dependent exchange of its monomers, and the model demonstrates that this monomer exchange allows the maintenance of robust oscillations. Finally, we perturbed the in vitro oscillator with temperature pulses to demonstrate the resetting characteristics of this unique circadian oscillator. Our study analyzes a circadian clockwork to an unprecedented level of molecular detail.

## Abstract 3

The interaction dynamics of the three-Kai-protein in vitro oscillator derived from the cyanobacterial circadian clock were determined to an unprecedented level of molecular detail.

## Introduction

Circadian clocks are self-sustained biochemical oscillators that underlie daily rhythms of sleep/waking, metabolic activity, gene expression, and many other biological processes. Their properties include temperature compensation, a time constant of approximately 24 h, and high precision. These properties are difficult to explain by known biochemical reactions. The ultimate explanation for the mechanism of these unusual oscillators will require characterizing the structures, functions, and interactions of the molecular components of circadian clocks. We are analyzing the components of the biological clock in the simplest cells that are known to exhibit circadian phenomena, the prokaryotic cyanobacteria, in which genetic and biochemical studies have identified three key clock proteins, KaiA, KaiB, and KaiC in the cyanobacterium Synechococcus elongatus. The atomic structures of these proteins have been determined, and a recent study reported the amazing observation that these three proteins can reconstitute a circadian oscillation in vitro, including its temperature-compensation property [1]. This in vitro circadian oscillator is the best available system for structural and biophysical analyses of a circadian clockwork.

A mutational analysis led to the discovery that the circadian clock in S. elongatus is regulated by at least three essential clock genes, *kaiA, kaiB,* and *kaiC,* that form a cluster on the chromosome [2]. The proteins encoded by these genes interact with each other and influence each other's activity [3–5]. KaiC appears to be the focal protein; it rhythmically oscillates between hypophosphorylated and hyperphosphorylated forms in vivo [6–8], and its phosphorylation status is correlated with the period of the clock [8]. KaiC autophosphorylates and autodephosphorylates in vitro [6,8]. KaiA and KaiB modulate the phosphorylation status of KaiC in vitro and in vivo: KaiA enhances KaiC's phosphorylation status, whereas KaiB antagonizes the effects of KaiA [7–10]. It may be that KaiA stimulates KaiC autophosphorylation directly [7,9,10], or it may enhance KaiC phosphorylation by inhibiting the autodephosphorylation of KaiC [8].

In 2004, we reported the crystal structure of the core protein of the circadian clock system from *S. elongatus,* the KaiC homohexamer, at 2.8-Å resolution [11]. In the same year, crystal structures for the KaiA and KaiB proteins were reported by other groups [12–14]. The KaiC structure resembles a double doughnut with 12 ATP molecules bound at the interfaces between subunits. The KaiCI and KaiCII domains of each subunit adopt similar conformations, but their ATP-binding pockets exhibit significant differences that are likely of functional importance. Previously identified KaiC mutations that affect rhythmicity and length of period include residues that map to the subunit interface. In contrast to conclusions of previous studies that suggested a single region of KaiA–KaiC interaction [5,13], the KaiC structure revealed the existence of two potential KaiA-binding regions, one mapping to the waist between CI and CII, the other to the dome-shaped surface presented by the CII hexamer. Together with the results from Vakonakis and LiWang [15], our recent results suggest that the most important binding site of KaiA to KaiC is on the surface of the CII terminus and to 22–amino acid “tentacles” that extend from CII [16]. Finally, the structure also identified T432, S431, and T426 in KaiCII as sites at which KaiC is phosphorylated [17]. The important role of these residues was confirmed by the loss of rhythmicity in T432A, S431A, and T426A single mutants [17].

The 2.0-Å crystal structure of KaiA from S. elongatus revealed that the protein is composed of two independently folded domains connected by a linker [14]; the C-terminal four-helix bundle domain has been shown to contain the KaiC-binding site [15]. The 2.2-Å crystal structure of KaiB from *Anabaena* revealed an alpha-beta meander motif [12,18]. Although the folds of KaiA versus KaiB are clearly different, their size and some surface features of the physiologically relevant dimers are very similar. Notably, the functionally important residues Arg69 of KaiA and Arg23 of KaiB align well in space. The apparent structural similarities suggest that KaiA and KaiB may compete for a potential common binding site on KaiC [12,18], and these similarities may explain KaiB's ability to antagonize the effects of KaiA upon KaiC phosphorylation [7–10]. The advent of atomic resolution structures of the molecular components of a clock marks a dramatic watershed in circadian clock research by ushering in truly molecular analyses of circadian mechanisms. Now the impact of mutations can be assessed in structural terms, and predictions of clock function and protein interactions can be addressed at an unparalleled level of molecular detail both in vitro and in vivo.

We are analyzing the structures, functions, and interactions of the KaiABC proteins by electron microscopic (EM) and crystallographic methods [11,16,17,19]. Based on the observations that (1) KaiA, KaiB, and KaiC interact with each other and these interactions modulate the status of KaiC phosphorylation [7–10], and (2) KaiC phosphorylation is rhythmic over the circadian cycle [7,20], it is reasonable to conclude that KaiA, KaiB, and KaiC form complexes whose composition oscillates over the circadian cycle. Using gel filtration of aliquots taken from the KaiABC in vitro oscillator, Kageyama et al. [21] have shown that there are cyclic changes in the amount of KaiA and KaiB that associate with KaiC. They also demonstrated that there is exchange of monomers between KaiC hexamers; this exchange increases during the KaiC dephosphorylation phase. We show in this study that the rhythmicity of KaiABC complexes can be visualized by EM and quantified by two-dimensional blue-native/sodium dodecyl sulfate gel electrophoresis (2-D BN/SDS-PAGE). We find that there is a mixture of types of Kai complexes at every phase in which the proportions of the various types oscillate. The data from this and other studies are used to derive a dynamic model for the in vitro oscillator that accurately predicts the rhythm of the KaiABC complexes. We also use fluorescence resonance energy transfer (FRET) with two populations of fluorescently labeled KaiC hexamers to confirm that monomer exchange among hexamers occurs. The model indicates that the function of this monomer exchange might be to maintain synchrony among the KaiC hexamers in the reaction, thereby sustaining a high-amplitude oscillation over multiple days. Finally, we apply the first perturbation experiments of the in vitro oscillator by temperature pulses to demonstrate that this unique circadian oscillator can be reset by stimuli that are well known to entrain circadian clocks.

## Results

### Rhythms of KaiABC Complexes Quantified by EM and Electrophoresis

**Figure 1:**
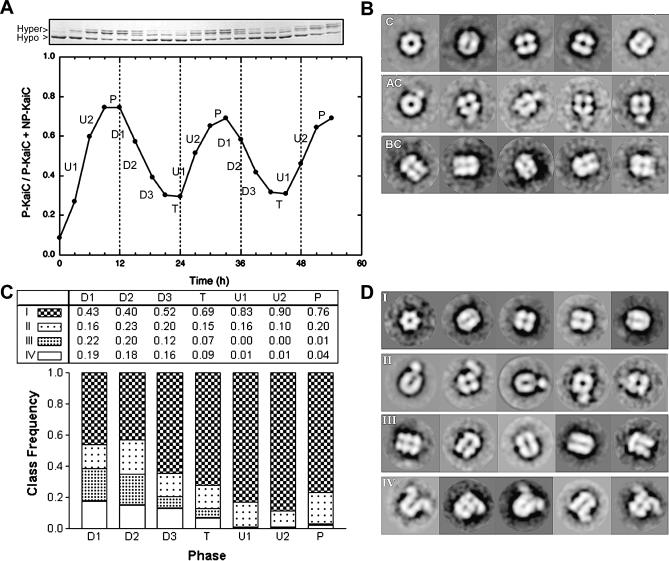
EM Analysis of the Temporal Sequence of KaiABC Complex Formation (A) The in vitro oscillation of KaiC phosphorylation (upper panel), in which the lower band is hypophosphorylated KaiC (NP-KaiC) and the upper bands are different forms of hyperphosphorylated KaiC (P-KaiC). The lower panel is a quantification of the ratio of P-KaiC to total KaiC (P-KaiC + NP-KaiC) as a function of time. The oscillation cycle is divided into seven phases for the negative-stain EM analyses in (C) and (D). (B) Representative EM class-sum images of KaiC alone (labeled C), KaiA•KaiC complexes (AC), and KaiB•KaiC complexes (BC). (C) Determination of the relative frequency of the four meta classes at each phase of the KaiABC cycling reaction. The table reports the meta-class frequencies (top), and is plotted in the histogram (bottom) for each phase (as defined in [A]). (D) Representative EM class-sum images generated for various phases of the KaiABC cycling reaction. The class-sum images were assigned to one of four structurally distinct meta classes (I–IV).

To track the temporal changes in the protein complexes formed during the in vitro circadian cycle, we performed a negative-stain EM analysis of samples drawn from the oscillating KaiABC mixture. At each phase of the in vitro oscillation of KaiC phosphorylation status (Figure 1A), there appeared to be a mixture of various macromolecular assemblies formed by the three Kai proteins. We used image classification, combined with comparison to EM class-sum images of defined KaiABC complexes, to sort individual particle images into four structurally distinct meta classes (see Materials and Methods for full description). Figure 1B shows EM class-sum images of KaiC alone or in pairwise combination with KaiA or KaiB to be compared with examples in Figure 1D of class-sum (average) images for each meta class (I–IV). These comparisons allowed us to assign the identity of Class I as KaiC hexamers alone, Class II as a KaiA•KaiC complex, and Class III as a KaiB•KaiC complex (compare Figure 1B with 1D). The molecular composition of Class IV has not been determined from EM data alone. (We will return to a probable identification of Class IV below.) The variation in percentage of each meta class was analyzed temporally during the KaiC phosphorylation cycle (Figure 1C). The data show that uncomplexed KaiC hexamers (Class I) are the predominant class at every phase. Class II (KaiA•KaiC) is approximately 15% of the total at every phase. During the phases in which the KaiC phosphorylation is decreasing (phases D1–D3), there are significantly larger percentages of Classes III and IV than at other phases of the cycle. Because we observe a mixture of several meta classes at each stage of the cycle, clearly KaiC hexamers do not simply progress from one type of complex to another at each phase of the in vitro oscillation, but rather exist as a population of different types of complexes at each phase (Figure 1C, also see Figure S1). This phenomenon will be modeled below.

**Figure 2:**
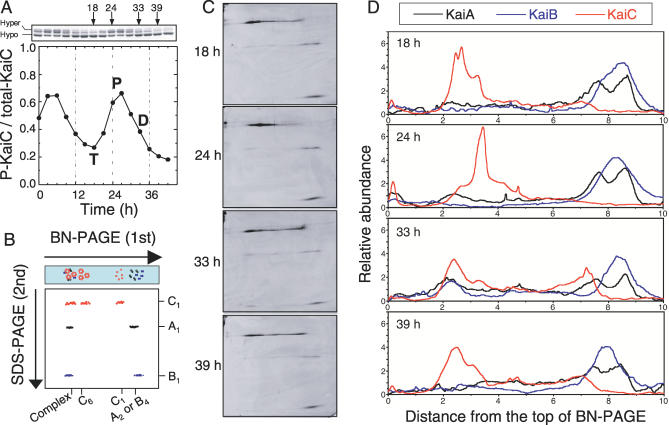
Two-Dimensional BN/SDS-PAGE Analysis of KaiABC Complexes from Different Phases of the In Vitro Oscillator (A) Rhythm of KaiC phosphorylation state of the in vitro KaiABC oscillator. At the time points indicated by arrows (18, 24, 33 and 39 h), an aliquot of the reaction was withdrawn and subjected to the 2-D BN/SDS-PAGE. Hyper, hyperphosphorylated; Hypo, hypophosphorylated. (B) Schematic illustration of the analysis of KaiABC complexes by 2-D BN/SDS-PAGE. A sample containing KaiABC proteins and their non-covalently bound homo-/hetero-oligomeric complexes was first subjected to BN-PAGE (∼4–6 °C for 10 h). Native protein complexes bound to the negatively charged Coomassie Blue dye were separated on a native gel (first dimension, from left to right). A lane of the BN-PAGE was excised, and subunits of the native complexes were separated by SDS-PAGE (second dimension, from top to bottom). (C) Coomassie Blue-stained BN/SDS-PAGE gels. KaiABC proteins from the in vitro cycling reaction at 18, 24, 33 and 39 h were separated by 2-D BN/SDS-PAGE. Gels were stained with colloidal Coomassie Blue. (D) Cyclic formation of KaiABC protein complexes. KaiABC proteins composing complexes were quantified by densitometry. Each trace shows the relative amount of KaiA (black), KaiB (blue), and KaiC (red) proteins. The horizontal axis represents the relative mobility of the proteins in their native state (0 = large mass, 10 = small mass).

Kageyama et al. [21] used gel filtration and immunoblotting of aliquots from different phases of the in vitro oscillation to analyze the relative changes in the interactions among KaiA and KaiB and KaiC. We report herein 2-D native electrophoresis of aliquots from different in vitro phases as an alternative method to quantify the complexes (Figure 2). Our technique used 2-D BN/SDS-PAGE [22–26], in which proteins are mixed with Coomassie Blue dye and electrophoresed in a native gel for the first dimension. The principle of this method is that the structure of complexes is preserved by the dye and that the electrophoretic mobility of a protein complex is determined by the negative charge of the bound dye and the size and shape of the complex. The resolution of BN-PAGE is higher than that of other methods such as gel filtration or sucrose-gradient ultracentrifugation [24]. The second dimension is then a standard SDS-PAGE, which allows the identification of the proteins by their apparent molecular weight. Using 2-D BN/SDS-PAGE, we obtained results that are consistent with the EM analysis in the multiple types of macromolecular assemblies that are present in each of the phases of the KaiABC oscillation. As shown in Figure 2, at all phases of the reaction, KaiC is found predominantly in the large-mass range (left side of the BN-PAGE), indicating that it is usually in hexameric form. The banding pattern for this high molecular weight KaiC distribution is bimodal, with the left-most peak indicating complexes of KaiC hexamers with KaiA and/or KaiB, and the right-most peak indicating free hexamers. The co-migration of KaiC hexamers with both KaiA and KaiB is most obvious at D2/D3 phases (especially at 33 h); this could be any combination of KaiA•KaiB•KaiC, KaiA•KaiC, or KaiB•KaiC. As indicated by the right-shifted KaiC hexamer peak and minimal co-migration of KaiB at 24 h, most of the KaiC is present as uncomplexed hexamers at P phase (24 h). There is also a small amount of KaiA•KaiC that co-migrates at all phases. Finally, at D2/D3 (33 h), there is a clear peak of KaiC monomers (between “6” and “8” on the abscissa of Figure 2D) that coincides with an increased rate of KaiC monomer exchange at this phase (see below and [21]). Neither 2-D BN/SDS-PAGE (Figure 2) nor gel filtration [21] detected a complex that is specifically KaiB•KaiC, but the Class III complex of the EM data strongly supports the existence of a KaiB•KaiC complex (Figure 1), and thereby confirms the data obtained by pull-down assays [21]. It is possible that the KaiB•KaiC complex is not resolved from other complexes under the conditions of electrophoresis or gel filtration, whereas negative-staining/EM preparation or immunoprecipitation can specifically identify the KaiB•KaiC complex.

### Modeling the In Vitro KaiABC Oscillator

**Figure 3:**
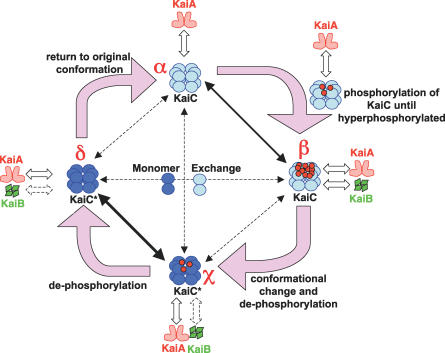
Diagram of the Model for the KaiABC Oscillator Depicted is a representation of the mathematical model for the phosphorylation cycle of a KaiC hexamer and its association with KaiA and KaiB. A KaiC monomer is shown as a double circle that forms a hexamer that can associate/dissociate with KaiA and/or KaiB. KaiC hexamers are depicted in both their default status (light blue color) and in an altered KaiC* state that has undergone a conformational change (darker blue color). Red dots are phosphates attached to phosphorylation sites on KaiC (residues T426, S431, and T432). KaiC hexamers can exchange monomers between hexamers in any of the states, depicted by the double-headed arrows in the central region of the figure. The relative rates of exchange between these states are illustrated by the type of double-headed arrows (thick line = high rate; thin line = medium rate; and dashed line = low rate).

Based on the data from the previous investigations of the in vitro KaiABC oscillator [1,21] and our data from EM and BN-PAGE analyses, we propose a model for this novel molecular oscillator (Figure 3). Briefly, we have explicitly modeled the kinetics of KaiC hexamers and the degree of phosphorylation of each monomer in every hexamer. Starting from a hypophosphorylated state of KaiC (state α), rapid association and disassociation of KaiA facilitates phosphorylation until the KaiC hexamer is hyperphosphorylated (state β). KaiB is assumed to bind with hyperphosphorylated KaiC; KaiB association with KaiC induces a simultaneous conformational change to a new state (KaiC*, state χ). The KaiC* hexamer (state χ) dephosphorylates to a relatively hypophosphorylated status (state δ) and relaxes to the original conformation (state α). Simultaneous with the phosphorylation cycle of a hexamer is the possibility of the exchange of monomers between any two hexamers in any of the states. The rates of this exchange may differ depending on the conformational state of the KaiC hexamers, their degree of phosphorylation, and their association with KaiA or KaiB (Figure 3).

To be more specific (see Text S1 for a full description of the model), we assume that KaiA stochastically binds and unbinds rapidly from KaiC hexamers. When KaiA is bound to KaiC (in state α), the monomers in the complex are phosphorylated at a faster rate than in the absence of KaiA, so that this protein acts as an amplifier of KaiC's intrinsic ability to autophosphorylate [7], and it inhibits KaiC's rate of dephosphorylation [8]. We assume that KaiB associates/disassociates with the KaiC hexamer when the total degree of phosphorylation of the hexamer exceeds a threshold that places KaiC in state β. Therefore, the interaction between KaiB and KaiC is contingent upon conformational changes resulting from hyperphosphorylation; this assumption is based on the observation that there is a preferential association of KaiB with hyperphosphorylated KaiC versus hypophosphorylated KaiC [21]. The model depicted in Figure 3 further assumes that binding of KaiB irreversibly alters the conformation of the KaiC hexamers (KaiC → KaiC* = state χ). The rates of binding/unbinding of KaiB to hyperphosphorylated KaiC are also rapid relative to the oscillation timescale [21]. The altered KaiC* hexamers may continue to form complexes with KaiA (in addition to KaiB). The model assumes that the KaiA•KaiC* (and KaiC*) complexes are essentially non-phosphorylatable or have a slower rate of phosphorylation than the rate of autodephosphorylation so that KaiA is no longer able to amplify KaiC phosphorylation. Finally, non-phosphorylated KaiC* (state χ) relaxes to the original non-phosphorylated KaiC state (state α).

**Figure 4:**
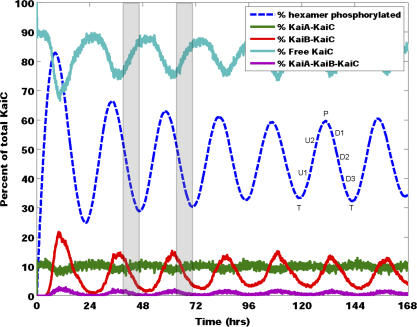
Model Prediction of KaiA/KaiB/KaiC Complexes as a Function of Time in the In Vitro Oscillation Reaction The dashed blue line is the rhythm of KaiC phosphorylation status, and the other lines are the predictions of the percentage of each of the indicated complexes, i.e., KaiA•KaiC, KaiB•KaiC, KaiA•KaiB•KaiC, and free KaiC. The grey shaded areas are the phases when the model predicts maximal monomer exchange. (These phases are shown for only two cycles, but they are characteristic of the dephosphorylation phases for all cycles.) Cycle #6 is also labeled with the phases defined in Figure 1A.

Based on the recent suggestion of monomer exchange among KaiC hexamers [21], the model allows for different rates of monomer exchange between KaiC-KaiC, KaiC-KaiC*, and KaiC*-KaiC* hexamers (double-headed arrows in the central region of Figure 3), i.e., between all the states in the model that are simplified in Figure 3 as α, β, χ, and δ. The relative rates of exchange between these states are illustrated by the type of double-headed arrows in Figure 3 (thick line = high rate; thin line = medium rate; and dashed line = low rate). The model can be used to predict the time dependence of the formation of complexes among the KaiA/KaiB/KaiC proteins. Figure 4 depicts these predictions. Uncomplexed KaiC hexamers (i.e., those that are not associated with KaiA or KaiB) form the largest fraction of the KaiC population at all phases. However, at approximately the time of peak KaiC phosphorylation, the percentage of free KaiC hexamers begins to drop, and KaiB•KaiC and KaiA•KaiB•KaiC complexes become a significant fraction of the total population during the dephosphorylation phases (Figures 3 and 4). The fraction of KaiA•KaiC complexes is relatively constant throughout the cycle. These predictions correspond closely to the experimental data obtained by the EM analyses (Figure 1C). The Class I complex is free KaiC hexamers; this complex forms the largest fraction and oscillates in the same phase relative to the rhythm of KaiC phosphorylation in both the model and the experimental data. The Class II complex (Figure 1D) appears to be the same as KaiA•KaiC (Figure 1B and see [16]), and the fraction of this complex is relatively constant throughout the cycle in both the experimental data (Figure 1C) and the model (Figure 4). The Class III complex appears to be the same as KaiB•KaiC, and these increase in percentage during the dephosphorylation phase (Figure 1C) as predicted by the model (Figures 3 and 4). We interpret the Class IV complex to be equivalent to the complex of all three proteins (KaiA•KaiB•KaiC) as described in the next section.

### Confirmation of Monomer Exchange with FRET

**Figure 5:**
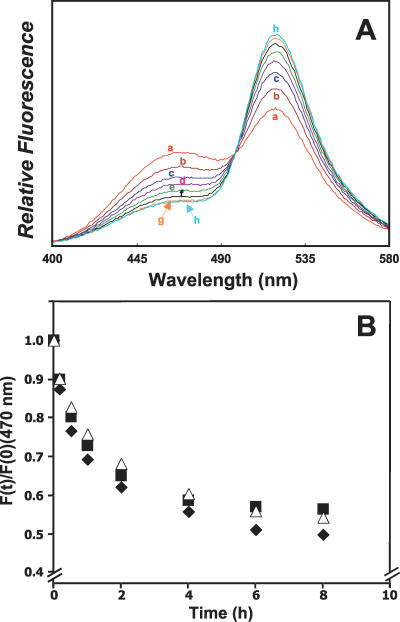
KaiC Monomer Exchange Revealed by Changes in FRET Signal (A) One sample of KaiC was labeled with IAEDANS, and another sample of KaiC was labeled with MTSF. The emission spectra of KaiC (0.2 μg/μl total concentration) excited at 336 nm was recorded at the following times: (a) 0, (b) 0.16, (c) 0.5, (d) 1, (e) 2, (f) 4, (g) 6, (h) 8 h after mixing equal amounts of IAEDANS-labeled and MTSF-labeled KaiC (0.1 μg/μl each) at 30 °C. The decrease in fluorescence intensity at 470 nm of IAEDANS-labeled KaiC is indicative of energy transfer due to monomer exchange between the two labeled KaiC populations. (B) Effect of KaiA and KaiB on monomer exchange. Measurement of monomer exchange between IAEDANS-labeled and MTSF-labeled KaiC when KaiA (0.05 μg/μl) or KaiB (0.05 μg/μl) was added the mixture of KaiC. The decrease in fluorescence intensity at 470 nm was plotted as a function of time. Symbols are KaiC alone (filled diamonds), KaiC + KaiA (filled squares), KaiC + KaiB (open triangles).

Kageyama and coworkers [21] used FLAG-tagged KaiC proteins to demonstrate that KaiC monomers appear to exchange, or “shuffle,” between KaiC hexamers. The rate of this exchange appeared to be maximal during the phase of KaiC dephosphorylation and was reported by Kageyama and coworkers to be inhibited by KaiA [21]. However, the pull-down assays of FLAG-tagged KaiC protein interaction may suffer from differential cross reactivity and aggregation. Therefore, we decided to avoid the use of peptide-tagged proteins by employing a completely different technique to confirm KaiC monomer exchange, namely FRET [27,28]. KaiC has three intrinsic cysteine residues that can be used for labeling with fluorophores. We used IAEDANS and MTSF, which are well-characterized FRET fluorophore partners. Therefore, one group of KaiC hexamers was labeled with IAEDANS and the other with MTSF. These groups were then mixed and incubated while monitoring the time-dependent change in quenching of IAEDANS fluorescence (indicative of FRET) in response to excitation at 336 nm (the excitation maximum for IAEDANS). As shown in Figure 5A, during the incubation of the two populations of KaiC, the emission at 470 nm (emission peak for IAEDANS) decreases progressively. This result indicates that monomer exchange among the two groups of KaiC has occurred so that the donor (IAEDANS) fluorescence is quenched. A change in the FRET ratio is obvious even after only 10 min, indicating that monomer exchange is a rapid event among KaiC hexamers. After 8 h of incubation, the quenching of IAEDANS fluorescence decreased by approximately 50% of the initial intensity. The FRET ratio does not change in KaiC preparations that are labeled with only MTSF (unpublished data). Unlike the results obtained with the previously reported pull-down assay [21], our FRET analysis indicates that neither KaiA nor KaiB significantly inhibits monomer exchange (Figure 5B).

The 2-D BN/SDS-PAGE data also provide support for the exchange of KaiC monomers among hexamers preferentially during the dephosphorylation phases of the reaction (Figure 2). The distribution of low molecular weight species of KaiC is different among the phases of the in vitro reaction with KaiC predominantly hexameric at T, U, and P phases. During the dephosphorylation phase, there is a significant amount of lower molecular weight KaiC (especially obvious at 33 h in Figure 2). This lower molecular weight KaiC might indicate the presence of KaiC monomers (or possibly dimers/trimers) that are a consequence of monomer exchange.

**Figure 6:**
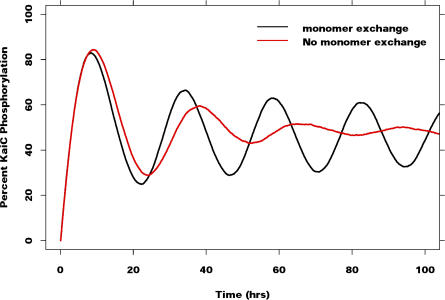
Model Prediction of the In Vitro KaiABC Oscillation Reaction in the Presence or Absence of Monomer Exchange When monomer exchange is allowed, the rhythm of KaiC phosphorylation is well sustained, but it dampens rapidly when monomer exchange is disallowed.

What are the potentially functional consequences of the monomer exchange? Kageyama and coworkers [21] suggest that monomer exchange may form the basis for KaiC dephosphorylation and/or may help to equalize dephosphorylation rates among KaiC hexamers. As recognized by Emberly and Wingreen [29], monomer exchange can synchronize a population of hexamers. In particular, monomer exchange endows the model with a crucial feature, that of synchronization among the KaiC hexamers in the population in the appropriate phase. This synchronization is probably not perfect, so we allow for the possibility of weak monomeric exchange among hexamers of the different phases. If the monomeric exchange between hexamers is not allowed during the simulation, then the oscillations in KaiC phosphorylation dampen out (Figure 6). Alternatively, if the monomeric exchange between all states of hexamers is modeled with equivalent rates, then the oscillations will also dampen out. Therefore, the simulation underscores the importance for sustained oscillations of “rapid” exchange of monomers *within* states C and C* (i.e., within α and β and within χ and δ) and a low exchange rate *between* states C and C* (i.e., between α/β and χ/δ). With this combination of exchange rates, our simulations show a sustained, high-amplitude oscillation pattern for KaiC phosphorylation (Figure 6) that is consistent with experimental observations.

Do our EM studies give any information on the monomer exchange reaction? In particular, we considered whether the Class IV complex observed by EM (Figure 1) is a “snapshot” of hexamers caught in the act of exchanging their monomers. The other likely possibility is that the Class IV complex is a complex of KaiA, KaiB, and KaiC. We favor the latter interpretation, namely that the Class IV complex is KaiA•KaiB•KaiC for two major reasons. First, the phases when Class IV complexes appear in the EM analysis correspond well with the 2-D BN/SDS-PAGE (Figure 2) and gel filtration [21] data for the co-migration of KaiA, KaiB, and KaiC. Second, the model predicts that the KaiA•KaiB•KaiC complex will be maximally present in the early phases of KaiC dephosphorylation, i.e., D1 and D2 (Figure 4); monomer exchange, on the other hand, should peak at the late KaiC dephosphorylation (D3) and trough (T) phases (Figure 3 and see grey shaded areas on Figure 4). Therefore, although this hypothesis is not proven, the data and modeling support the conclusion that—at a minimum—a subpopulation of Class IV complexes are KaiA•KaiB•KaiC complexes.

### Testing the Model by Perturbation Analyses

An important analytical method for oscillators is by perturbation analysis. One type of perturbation that can be performed on this in vitro oscillator is by varying the concentration and/or stoichiometry of the Kai proteins. Kageyama and coworkers reported that diluting all three proteins caused the oscillation to stop at *low* values of KaiC phosphorylation [21]. In addition, reducing [KaiA] or [KaiB] alone while maintaining the concentration of the other two proteins had distinct consequences; reducing [KaiA] caused first a lengthening of period, and further dilution caused arhythmicity at *low* values of KaiC phosphorylation, whereas reducing [KaiB] below a critical concentration caused arhythmicity at *high* values of KaiC phosphorylation [21]. These experimental results are accurately simulated by our model (see Figure S4 and accompanying description).

**Figure 7:**
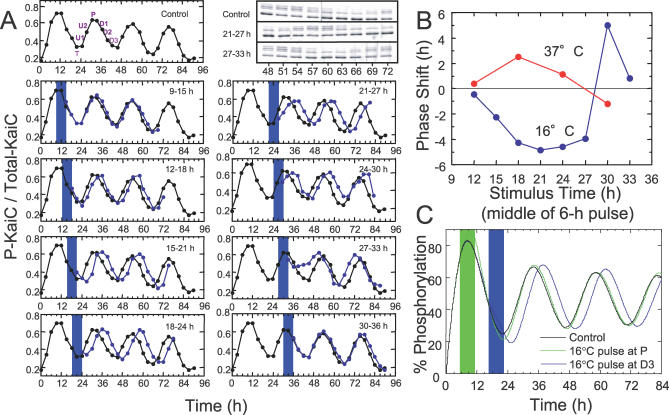
Perturbation of the Phase of the In Vitro KaiABC Oscillator by 6-h Exposures to 16 °C or 37 °C Administered at Different Phases of the Reaction Maintained at 30 °C (A) Raw data for 16 °C pulses administered at eight different phases (shaded blue areas indicate the time of the 16 °C pulse). Black symbols and lines are control (untreated) samples; blue symbols and lines are from the treated samples. The inset at the upper right shows the raw SDS-PAGE data for hours 48–72 for three samples: control, 16 °C pulse at hours 21–27 (resulting in a phase delay), and 16 °C pulse at hours 27–33 (resulting in a phase advance). (B) PRC of the 16 °C pulse data shown in (A) (blue) as well as phase shifts from high temperature pulses (red: 6-h pulse exposure to 37 °C). (C) Computational simulation of temperature-dependent phase shifts for 16 °C pulses administered at the peak (P) phase or at late dephosphorylation (D2) phase. For both pulses, the temperature reduction from 30 °C to 16 °C during the pulse is approximated by (1) an instantaneous reduction in KaiB-off and KaiA-off rates (to 1% of the 30 °C value), (2) a decrease in the monomer phosphorylation and dephosphorylation rates (assuming a *Q_10_* of 1.1), and (3) a decrease in the rate of relaxation from state δ to state α (to 10% of the 30 °C value). See Text S1 for details.

In the case of circadian oscillators, perturbation analysis has frequently taken the form of assaying the phase responsiveness of a stimulus, as in provoking “phase shifts” that can be plotted as phase response curves (PRCs, [30,31]). We therefore decided to also undertake a perturbation analysis of this in vitro oscillator using temperature-pulse stimuli to test whether this unique circadian oscillator can be reset by stimuli that are well known to entrain circadian clocks. As shown in Figure 7A, phase-dependent resetting of the in vitro oscillator occurs in response to a 6-h temperature shift from 30 °C to 16 °C. When KaiC phosphorylation is compared at the beginning and the end of the 6-h pulses to that of the control samples (run continuously at 30 °C), it appears that exposure to the 16 °C conditions slowed the rate of phosphorylation (U phases) or dephosphorylation (D phases), but did not stop those reactions. It is particularly interesting that KaiC phosphorylation during U phases continues during the exposure to 16 °C (e.g., see pulses at 21–27, 24–30, and 27–33 h). The impact of the 16 °C pulses on phase was dependent upon the timing of the exposure. The low-temperature pulses do not provoke significant phase shifting at peak phosphorylation phases (e.g., 9–15 or 30–36 h). There are delays of varying magnitudes for pulses that start at D1, D2, D3, T, and U1 phases, whereas pulses that begin at U2 phases (27–33 h) elicit a phase advance (Figure 7A). Figure 7B illustrates these phase shifts plotted as a PRC [30,31]. We also experimentally tested the effect of 37 °C pulses on the KaiABC oscillator and found phase shifts that mirror those of the 16 °C pulses—at phases when low-temperature pulses cause delay shifts, the high-temperature pulses cause advances, and vice versa (Figure 7B).

We examined our model for potential targets of temperature-pulse resetting and found that assuming the temperature reduction from 30 °C to 16 °C during the pulse causes (1) a reduction in KaiB-off and KaiA-off rates, (2) a decrease in the monomer phosphorylation and dephosphorylation rates (but retaining a temperature-compensated *Q_10_* of 1.1), and (3) a decrease in the rate of relaxation (from state δ to state α) allowed an acceptable simulation of the effect of low-temperature pulses on phase. In particular, a simulated 16 °C pulse centered at the time of peak KaiC phosphorylation does not elicit a significant phase shift, whereas a simulated 16 °C pulse centered near the end of dephosphorylation leads to a substantial phase delay (Figure 7C). Importantly, this simulated effect of temperature will not significantly affect the period of the model oscillator under continuous exposure to temperatures in the range of 25–35 °C (unpublished data). Thus the model allowed an acceptable simulation of the effects of low-temperature pulses on phase while retaining temperature compensation of the period.

## Discussion

### Precession of Kai Protein Complexes during the In Vitro Oscillation

As predicted from the results of previous studies, the rhythm of KaiC phosphorylation in vitro is accompanied by rhythmic association patterns among the KaiA/KaiB/KaiC proteins. Our EM and BN-PAGE analyses have allowed the elucidation of the precession of Kai protein complexes over the circadian cycle of the in vitro oscillator. Both methods indicate that there is not a single species of complex present at each phase. Also, both EM and BN-PAGE demonstrate that the in vitro oscillation cycle does not proceed with a single species of the complex proceeding in lock step to another species of complex at the next phase. Rather, there is a mixture of types of Kai complexes at every phase, in which the proportions of the various types oscillate. Free KaiC hexamers and KaiA•KaiC complexes are present throughout the cycle. During the KaiC dephosphorylation phases (D1, D2, D3, and T), KaiB is also associated with KaiC as both KaiB•KaiC and KaiA•KaiB•KaiC complexes (Figures 1 and 2). In the dephosphorylation phases, a significant amount of monomeric KaiC is also present (Figure 2), which corresponds to the phase at which monomeric exchange is expected. Our results using EM and BN-PAGE are in good agreement with the previous results obtained by gel filtration and pull downs [21]; however, our higher-resolution methods allow quantification of the different proportions of the complexes, and the EM studies in particular allow the visualization of the clockwork in action. Using FRET, we have been able to confirm that KaiC hexamers undergo monomer exchange. However, based on a pull-down assay with peptide-tagged protein, Kageyama and coworkers [21] reported that KaiA inhibits the KaiC monomer exchange rate. In contrast, our FRET assay does not find a significant effect of either KaiA or KaiB on KaiC monomer exchange. We do not currently understand the basis for this discrepancy, but we believe the FRET method is more reliable than the pull-down method since (1) the size of the fluorophores is smaller than that of the FLAG tags and (2) the FRET method does not depend upon a precipitation step for assay.

Although our FRET method suggests that KaiA does not affect the rate of KaiC monomer exchange, our EM data indicate that the level of KaiA/KaiC association (Class II complex) is relatively constant over the circadian cycle (Figure 1C). In contrast, the association of KaiB with KaiC (Class III) is clearly rhythmic. On the basis of similar regions in the three-dimensional crystal structures of KaiB and KaiA, Garces and coworkers [12] proposed that the antagonistic action of KaiB on KaiA is due to a shared binding site on the KaiC hexamer. However, if the Class IV complex reported herein is truly the visualization of a complex in which KaiA and KaiB and KaiC are simultaneously present, then it seems likely that KaiB does not act by a direct displacement of KaiA from a common binding site. This observation suggests a more complicated mechanism for KaiB's antagonism of KaiA's activity. Conformational changes appear evident in the Class IV complex. It is possible that KaiB's inclusion in the Class IV complex alters KaiA's activity such that it is no longer productive in stabilizing a hyperphosphorylated state of KaiC. The data of Figure 1C that show a relatively constant level of KaiA•KaiC throughout the circadian cycle raises the question, “Why does a relatively constant level of KaiA•KaiC complex not maintain a stably non-rhythmic population of hyperphosphorylated KaiC?”

### Modeling of the In Vitro Oscillator

Our modeling of the in vitro oscillator provides a potential answer to this question and offers other important insights into the dynamics of the circadian clock. Modeling may suggest explanations for the existence and absence of oscillations under varying conditions in vitro that are potentially relevant in vivo, and thereby assist in experimentally answering fundamental questions related to the construction and functional properties of this unique molecular oscillator. To address these questions, we have simulated the biochemical interactions of the Kai proteins and kept track of the degree of phosphorylation of each monomer on every KaiC hexamer in biochemical simulations (the number of KaiC hexamers monitored in typical simulations is approximately 10^3^–10^4^). The model is capable of generating realistic complex kinetics and sustained oscillations in KaiC phosphorylation using experimentally motivated interactions. As depicted in Figure 4, this model does an excellent job of matching the proportions of the different KaiA/KaiB/KaiC complexes observed in the EM and BN-PAGE data (Figures 1 and 2) as well as the previously reported gel filtration and pull-down data [21].

The salient features of the experimental findings have an obvious interpretation using the model simulations as a guide. Because KaiB is assumed to associate and disassociate only from hyperphosphorylated KaiC (state β, [21]), the maximal rate of KaiB association with KaiC occurs around the time of peak KaiC phosphorylation (Figure 4). Therefore, the peak of KaiB–KaiC complex formation peaks near the KaiC phosphorylation peak, but is slightly delayed due to a slight asynchronicity in the population of hyperphosphorylated KaiC hexamers. In the model, we assumed that the association of KaiB alters the conformation of KaiC into a new, stable conformational state (KaiC*, states χ and δ of Figure 3) in which dephosphorylation occurs more rapidly than phosphorylation. Therefore, we can interpret the subsequent phase of dephosphorylation (state χ of Figure 3) as a direct result of this conformational change. The apparently symmetric profiles of the peaks in the KaiC phosphorylation rhythm is then interpreted as similar effective rates for dephosphorylation (in the KaiC*, KaiA•KaiC* state) versus phosphorylation (in the KaiA•KaiC state). In the model, KaiA rapidly associates with KaiC and dissociates from KaiC at every phase, but when it binds to hypophosphorylated KaiC (state α), KaiA facilitates KaiC autophosphorylation so that it can transition to the hyperphosphorylated (state β), which binds to KaiB and restarts the cycle (Figure 3). The relative percentage of KaiA•KaiC complexes (∼10%–15%) is reproduced in simulations as a result of faster disassociation of KaiA from KaiC versus association. In the absence of KaiB, the increase to approximately 85%–90% phosphorylation of KaiC from a hypophosphorylated state—despite only approximately 10%–15% KaiA bound at any time—is achieved through a separation of timescales between these KaiA•KaiC interactions (“fast”) versus phosphorylation/dephosphorylation rates (“slow”).

In the model, monomer exchange is shown to directly provide a mechanism for synchronizing the number of phosphates on different hexamers of a given conformation. Therefore, the oscillation in the phosphorylation rhythm of the hexamer population proceeds in a regulated fashion. The rates of this exchange may differ depending on the conformational state of the KaiC hexamers, their degree of phosphorylation, and their association with KaiA or KaiB (Figure 3). Monomer exchange among KaiC hexamers is now confirmed by both FLAG-tag pull downs [21] and FRET (Figure 5). Different hypotheses have been suggested for the role of monomer exchange. For example, Kageyama and coworkers suggested that monomer exchange “of KaiC hexamers may be a potential dephosphorylation mechanism” [21]. Even before Kageyama and coworkers [21] reported the existence of KaiC monomer exchange, Emberly and Wingreen [29] anticipated the possibility of KaiC monomer exchange and indicated that such exchange was sufficient to synchronize the phosphorylation of a population of KaiC hexamers. However, Emberly and Wingreen [29] also hypothesized that KaiC might form extended clusters of hexamers during the dephosphorylation phase, which we do not observe by EM. Therefore, our hypothesis at the present time is that KaiC monomers (or dimers or trimers) exchange into hexamers as individual events rather than as part of long, extended strings of KaiC hexamers merging and unmerging. As shown in Figure 6, our model underscores the potential significance of monomer exchange in the synchronization of KaiC-containing complexes and therefore in sustaining high-amplitude oscillations. Therefore, our current interpretation of the data is that monomer exchange serves to synchronize KaiC hexamers within the population as suggested by Emberly and Wingreen [29], but not via the merging of large, extended complexes. It is also possible that changes in the rate of monomer exchange could be responsible for phase resetting by temperature and/or light stimuli. Finally, Kageyama and coworkers [21] reported that KaiA inhibits the KaiC monomer exchange rate significantly, but this effect does not appear to be significant when monomer exchange is assayed by FRET (Figure 5). As described above, we favor the FRET method for assaying KaiC monomer exchange; however, our model oscillator is not sensitive to this difference—whether KaiA inhibits or does not inhibit KaiC monomer exchange does not significantly affect the behavior of the model (see Text S1).

### Perturbation Analyses of an In Vitro Oscillator

The temperature-pulse PRC data depicted in Figure 7 is the first example of a pulsatile perturbation analysis of an in vitro oscillator. In this case, we used a stimulus that is well documented to be an entraining agent of in vivo circadian oscillators [31,32]. As expected, pulses lead to phase-dependent resetting of the in vitro clockwork. The explanation of how an oscillator can be *period*-compensated for different constant temperatures while still *phase*-sensitive toward temperature transitions (as in temperature pulses) is not trivial. As a first step towards identifying potential temperature-sensitive steps that could account for the temperature-pulse resetting while allowing temperature compensation of the period, we examined which rates in the model have the largest impact on oscillating behavior. We found that modulations of the rates of KaiC phosphorylation and KaiC dephosphorylation have the largest effects on period and phase in our model. However, experimental data indicate that these rates are well temperature compensated ([20] and unpublished results), so we looked elsewhere for a potential target of temperature-pulse resetting. We therefore set the rates for KaiC phosphorylation and dephosphorylation to be temperature compensated in the model and explored other rates as potential resetting targets. An acceptable simulation of the effect of low-temperature pulses on phase was attained by assuming the temperature reduction from 30 °C to 16 °C during the pulse caused (1) an instantaneous reduction in KaiB-off and KaiA-off rates, (2) a decrease in the monomer phosphorylation and dephosphorylation rates (assuming a *Q_10_* of 1.1), and (3) a decrease in the rate of relaxation from state δ to state α. This simulation of the effect of low temperature on the model oscillator did not significantly affect the period as exposed to different constant temperatures (Figure 7C). This simulation does *not* mean that the changes in these particular rates are the mechanism of temperature-pulse resetting, merely that it is possible to find parameters in our model that allow temperature transitions to elicit phase shifts without losing temperature compensation of the period. Therefore, the goal of this experiment and simulation is to show that phase-dependent resetting is possible with the in vitro oscillator and that reasonable assumptions can model this phase-shifting phenomenon.

### Relationship of the In Vitro Oscillator to the Entire Circadian System In Vivo

The circadian rhythm of KaiC phosphorylation is maintained in vivo when cells are transferred to constant darkness (DD)—a condition in which transcription and translation are inhibited in S. elongatus [20]. This observation indicates that circadian oscillations are possible in cyanobacteria without the operation of a transcription-translation feedback loop (TTFL). The in vitro KaiABC oscillator serves as an excellent model for the in vivo cyanobacterial clock in DD [1]. However, when cells are in constant light (LL) or in light/dark cycles (LD), there are global rhythms of transcription and translation that are mediated by the KaiABC oscillator [2,8,20,33,34]. Under LL and LD, there are oscillations in the abundance of KaiB and KaiC [20,34], and these changes may influence the nature of the fundamental oscillator, because studies of the in vitro KaiABC oscillator show that low levels of KaiB and KaiC cannot sustain rhythmicity [21]. Therefore, an outstanding issue of considerable interest is how the cyanobacterial circadian system is changed in LL/LD to include molecular turnover (transcription and translation and protein degradation) and whether a TTFL is necessary under these conditions.

The enzymatic activity of the KaiABC system in regulating circadian output rhythms in vivo is not known. KaiC shows structural similarities to helicases and recombinases, and it binds with low affinity to DNA [19]; KaiC has therefore been postulated to directly mediate rhythms of DNA torsional stress that could regulate transcription globally [35,36]. Another hypothesis for the action of KaiC that is not mutually exclusive is that KaiC can promote the autophosphorylation of the two-component histidine kinase SasA, which in turn phosphorylates a response regulator RpaA that is essential for the mediation of rhythmic gene expression [37]. RpaA includes DNA-binding motifs in its sequence. Therefore, it is probable that KaiC impacts DNA either directly [35,36] and/or indirectly through SasA and RpaA [37], thereby regulating global gene expression [33].

Presently, the circadian clock system in cyanobacteria differs in a number of ways from that of eukaryotes [35]. Nevertheless, the cyanobacterial clock system in LL/LD is possibly a TTFL as in eukaryotes, whereas the DD system is purely post-translational. Might it be possible that the eukaryotic clock systems include an underlying timing circuit that is also totally post-translational? If so, perhaps there is a technique by which such an underlying post-translational clock can be “teased out.” If this tantalizing suggestion is ultimately found to be true, then investigations of the molecular mechanisms of the cyanobacterial clock system will have led to insights that are more broadly applicable to circadian clock systems of other organisms. Whether or not eukaryotic clocks will be found to have a purely post-translational mode, the discovery of the in vitro circadian oscillator composed of KaiA, KaiB, and KaiC [1] enlarges our understanding of how a circadian clockwork *can* be put together. Moreover, this study and that of Kageyama and coworkers [21] together with the structural investigations of Kai proteins and complexes [11–19] catapult circadian analyses to a truly molecular and biophysical level.

## Materials and Methods

### Preparation of Kai proteins and in vitro reactions.

Kai proteins from S. elongatus were expressed in Escherichia coli and purified as described by Nishiwaki and coworkers [38] with some minor modifications. In vitro reactions contained 20 mM HEPES-NaOH, (pH 8.0), 150 mM NaCl, 5 mM MgCl_2_, 0.5 mM EDTA, 1 mM ATP, 50-ng/μl KaiA, 50-ng/μl KaiB, and 200-ng/μl KaiC. The reaction mixture was dialyzed against this reaction buffer from which ATP was excluded at 30 °C for 24 h. The reactions were initiated by addition of 1 mM ATP and incubated in a 0.5-ml or 1.5-ml microcentrifuge tube (Sarstedt, Nümbrecht, Germany) at 30 °C in a circulating water bath. To determine the phosphorylation state of KaiC, an aliquot of the reaction was withdrawn every 3 h, mixed with SDS-PAGE sample buffer, and stored at −20 °C. Hyperphosphorylated and hypophosphorylated KaiC proteins were separated by SDS-PAGE (10% acrylamide). Gels were stained with colloidal Coomassie Brilliant Blue, and gel images were digitally captured by Bio-Rad Gel Doc XR system (Bio-Rad, Hercules, California, United State). For the pulse experiments, gels were stained with SYPRO Ruby (Invitrogen, Carlsbad, California, United States) and scanned with Typhoon (GE Healthcare, Chalfont St. Giles, United Kingdom). Digital images were analyzed by ImageJ (http://rsb.info.nih.gov/ij/).

### EM and sample preparation.

ATP was added to purified KaiA, KaiB, KaiC protein mixtures (time 0) and then held at a constant 30 °C in a circulating water bath for 48 h. Two samples were extracted every 3 h for negative stain and SDS-PAGE analysis. Samples for SDS-PAGE analysis were flash frozen in liquid nitrogen, and samples for negative staining were absorbed to a solid carbon surface and stained with 0.75% uranyl formate. Digital micrographs were collected with a 2 K × 2 K Gatan UltraScan 1000 camera (Gatan, Pleasanton, California, United States) on an FEI Tecnai-12 electron microscope (FEI Company, Hillsboro, Oregon, United States) operating at 120 keV with a nominal magnification of 67,000×, which results in an actual magnification of 98,000× at the level of the CCD camera. Digital micrographs have a pixel size of 1.5 Å on the molecular scale, and were binned during initial processing to have a 3-Å pixel. Micrographs were collected with a defocus range of −0.7 to −1.5 μm.

### Image processing.

Particles were visually selected and cropped from the micrographs using the BOXER routine in the EMAN software package [39]. Particle images were pooled from multiple experiments based on the KaiC phosphorylation state into seven phases (Figure 1A). Particle image stacks were created for each phase: D1 (14,072 particle images), D2 (8,344 particle images), D3 (11,665 particle images), T (8,445 particle images), U1 (11,650 particle images), U2 (8,256 particle images), and P (7,317 particle images). The IMAGIC-5 software package was used to filter the particle images to 15-Å resolution and translationally align them to a total-sum image [40]. Multivariate statistical analysis (MSA) of the particle images was used to generate initial class-sum images for each phase. Subsequent translational and rotational alignment of the particle images was performed with multi-reference alignment (MRA). In the final analysis, 250 class-sum images were generated for each of the seven phases after multiple rounds of image classification.

### Meta-class sorting.

In an attempt to assess the compositional nature of the complexes found within the KaiABC oscillation samples, we performed additional negative-stain EM analyses of KaiC alone, KaiA mixed with KaiC, and KaiB mixed with KaiC for comparison. Our EM analysis of KaiC alone and KaiA•KaiC complexes from two different cyanobacterial species has been published [16]. In that study, we found a more stable KaiA•KaiC complex for proteins from Thermosynechococcus elongatus compared to *S. elongatus*. Thus, we used the T. elongatus KaiA•KaiC EM reconstruction for the meta-class sorting procedure. Our published study [16] included determination of three-dimensional EM reconstructions of KaiC and KaiA•KaiC at 24-Å resolution. In this study, we exclusively used Kai proteins from S. elongatus. In order to generate the KaiB•KaiC complex, S. elongatus KaiB and KaiC samples were mixed, incubated at 30 °C, and examined by EM at various time points. After a 6-h incubation time, greater than 50% of the particles had a tri-layered appearance and were interpreted as KaiB•KaiC complexes. Approximately 4,000 particle images of negatively stained KaiB•KaiC complexes were collected, and a three-dimensional reconstruction was calculated at approximately 20-Å resolution.

Two-dimensional projections were generated (with a 10° angular spacing) for each of the three reference structures (KaiC, KaiA•KaiC, and KaiB•KaiC). The IMAGIC Euler-Anchor routine was used to determine Euler angles for each class-sum image of each of the seven KaiABC oscillator phases with respect to each of three reference structures. The results were sorted by the cross-correlation value reported by the Euler-Anchor routine. The Euler angles found for the KaiABC oscillator class-sum images were used to generate matching projections of each reference structure for visual comparison. A meta class (I–IV) was assigned for each class-sum image on the basis of both the cross-correlation values and the visual comparison with the reference projections. Three rounds of meta-class sorting were performed, with the final round utilizing three-dimensional reconstructions generated for meta classes I–IV as the reference structures (see Figure S2 and Text S1 for additional details).

### 2-D BN-PAGE and SDS-PAGE of KaiABC complexes from different phases of the in vitro oscillator.

BN-PAGE was performed on aliquots from different phases of the KaiABC in vitro oscillator by modification of previously described methods [22–26]. At each phase, 10 μl of the reaction was withdrawn, mixed with 2.5 μl of 50% glycerol at room temperature, immediately snap-frozen in liquid nitrogen, and then stored at −80 °C. The samples were thawed in an ice-water bath for 70–90 s, mixed with 1.35 μl of loading buffer (5% Coomassie Brilliant Blue G-250 [Serva Electrophoresis, Heidelberg, Germany], 0.5 M 6-aminohexanoic acid, and 100 mM Bis-Tris, [pH 7.0]), and immediately loaded on to a NuPAGE Novex 4%–20% Bis-Tris gel (1-mm thickness, 8 cm × 8 cm; Invitrogen) while electrophoresis was run at 100 V at 4–6 °C using 50 mM Tricine, 15 mM Bis-tris, 0.5 mM EDTA, 5 mM MgCl_2_, 1 mM ATP, 0.02% Coomassie Brilliant Blue G-250, (pH 7.0), as cathode buffer, and 50 mM Bis-tris, 0.5 mM EDTA, 5 mM MgCl_2_, (pH 7.0), as anode buffer. After loading the samples, the electrophoresis was continued for 10 h. After the electrophoresis, the lanes of BN-PAGE gel were excised and incubated in 2× SDS-PAGE sample buffer containing 4% SDS and 200 mM DTT for 30 min at room temperature. The denatured BN-PAGE slice was placed onto the top of the stacking gel of the SDS-PAGE gel (1.5-mm thickness, 15% acrylamide in resolving gel) and mounted with 0.7% agarose in stacking buffer with SDS. Electrophoresis was performed at room temperature. After the SDS-PAGE, gels were stained with colloidal Coomassie Brilliant Blue and scanned by Odyssey infrared imaging system (LI-COR, Lincoln, Nebraska, United States) in the 700-nm channel and then analyzed by Odyssey analysis software.

### Fluorophore labeling of KaiC and measurement of FRET.

The fluorophores used to label the KaiC protein were IAEDANS (Ex 336/Em 470 nm) and MTSF (Ex 490/Em 515 nm) (see Figure S3 for fluorescence spectra of IAEDANS and MTSF). 5-((((2-iodoacetyl)amino)ethyl)amino)naphthalene-1-sulfonic acid (1,5-IAEDANS) was purchased from Molecular Probes (catalog # I14; Eugene, Oregon, United States). 2-{(5-Fluoresceinyl)aminocarbonyl}ethyl Methanethiosulfonate-4-Fluorescein (MTSF), was purchased from Toronto Research Chemicals (catalog # F510000; North York, Ontario, Canada). KaiC in DTT-containing buffer (150 mM NaCl, 1 mM DTT, 1 mM ATP, 50 mM Tris-Cl, 5 mM MgCl_2_, [pH 8.0]) was desalted on a G-25 Sephadex desalting column (GE Healthcare) equilibrated with the same buffer except without DTT. After desalting, KaiC was incubated with a 10-fold molar excess of either IAEDANS or MTSF in the dark. The reactions were allowed to proceed for 2 h at room temperature and then overnight at 4 °C. Unreacted fluorophores were separated from the fluorescently labeled KaiC proteins with another G-25 Sephadex desalting column. FRET was employed to determine the monomer exchange between KaiC hexamers. The exchange reaction was initiated by mixing equal volumes of IAEDANS-labeled KaiC with MTSF-labeled KaiC in the same buffer that was used for desalting, and then placed in a stoppered cuvette at 30 °C (final concentrations of each labeled KaiC were 0.1 μg/μl for a final total KaiC concentration of 0.2 μg/μl). At time points of 0, 10, 30, 60, 120, 240, 360, and 480 m, the emission spectrum of the sample that was excited at 336 nm was recorded using a spectrofluorometer (QuantaMaster-7/2005 SE; Photon Technology International, Birmingham, New Jersey, United States). The decay of the emission at 470 nm (emission peak for the donor IAEDANS) was calculated and plotted as a function of time.

### Modeling.

The model was implemented in Matlab (The MathWorks, Natick, Massachusetts, United States) and Fortran (G77; Free Software Foundation, Boston, Massachusetts, United States) using Monte Carlo methods [41], and is available upon request (from MB). See Text S1 for simulation details and model parameters used in the figures.

## Supporting Information

Figure S1 Negative-Stain Electron Micrographs of KaiABC Oscillation Samples at Seven Phases of the KaiC Phosphorylation Reaction Approximately 800 electron micrographs were collected for each phase of the reaction. The sample aliquots were stained with 0.75% uranyl formate. Digital micrographs were collected with a Gatan UltraScan 1000 camera on an FEI Tecnai-12 electron microscope operating at 120 keV with a nominal magnification of 67,000×. Micrographs were collected with a defocus range of −0.7 to −1.5 μm. Selected areas are shown from representative micrographs of each phase: (A) D1 phase, (B) D2 phase, (C) D3 phase, (D) T phase, (E) U1 phase, (F) U2 phase, and (G) P phase. The scale bar represents 250 Å.
(791 KB PDF)

Figure S2 Comparison of KaiABC Oscillation Class-Sum Images with Meta Class I, II, III, and IV Projection Images Three rounds of classification and meta-class sorting were performed on the negative-stain EM particles images for seven phases of the KaiC phosphorylation oscillation reaction. The first round utilized three reference EM reconstructions of KaiC, KaiA•KaiC, and KaiB•KaiC. The second round involved re-classification of the particle images assigned to particular meta classes into new class-sum images and re-sorting into meta classes. The third round utilized EM reconstructions generated for meta classes I–IV as the reference structures. This figure shows eight representative KaiABC oscillation class-sum images along with matching projection images of the four meta-class reconstructions.
(A) Class-sum images assigned to meta class I most closely match projections of the meta class I reconstruction.
(B) Class-sum images assigned to meta class II most closely match projections of the meta class II reconstruction.
(C) Class-sum images assigned to meta class III most closely match projections of the meta class III reconstruction.
(D) Class-sum images assigned to meta class IV most closely match projections of the meta class IV reconstruction. There was some ambiguity in the meta-class assignment, but during the third round of sorting the majority (>80%) of the KaiABC oscillation class-sum images could be confidently assigned to one meta class.
(430 KB PDF)

Figure S3 Spectral Properties of IAEDANS-Labeled and MTSF-Labeled KaiC for FRET Experiments Excitation (solid line) and emission (dashed line) spectra of KaiC labeled with the donor fluorophore IAEDANS (top panel) or the acceptor fluorophore MTSF (bottom panel). The emission maxima for IAEDANS-labeled KaiC excited at 336 nm is 470 nm. The maximum excitation and emission spectral peaks for MTSF are 490 nm and 515 nm, respectively. However, there is some direct emission at 515 nm from MTSF-labeled KaiC when it is excited at 336 nm. Therefore, we used donor quenching as an indicator of resonance energy transfer in this paper rather than the 515:470 ratio because donor quenching is not influenced by the direct excitation of the acceptor fluorophore.
(43 KB PDF)

Figure S4 Simulated Kai-Concentration Perturbation Behavior on KaiC Phosphorylation Dynamics (A) The results of varying KaiA (2/3, 1/10, or 1/100), holding KaiC and KaiB fixed.
(B) The results of varying relative KaiB levels (1/10 or 1/100), holding KaiA and KaiC fixed.
(C) The results of varying KaiA, KaiB, and KaiC simultaneously (1/20, 1/50, or 1/100).
The control parameters are those of Figure 4 in the manuscript.
(9.0 MB TIF)

Text S1 Description of Mathematical Model and Further Information on EM and FRET Methodology (79 KB DOC)

## Abbreviations

2-D BN/SDS-PAGE: two-dimensional blue-native/sodium dodecyl sulfate polyacrylamide gel electrophoresis
EM: electron microscopy
FRET: fluorescence resonance energy transfer
LD: light/dark cycle
LL: constant light cycle
PRC: phase response curve
