# Supplementary material for: Elucidating the Ticking of an In Vitro Circadian Clockwork
Source: PLoS Biol. 2007 Mar 27;5(4):e93. doi: 10.1371/journal.pbio.0050093 (PMC1831719; doi:10.1371/journal.pbio.0050093)

Supplementary Figure 1

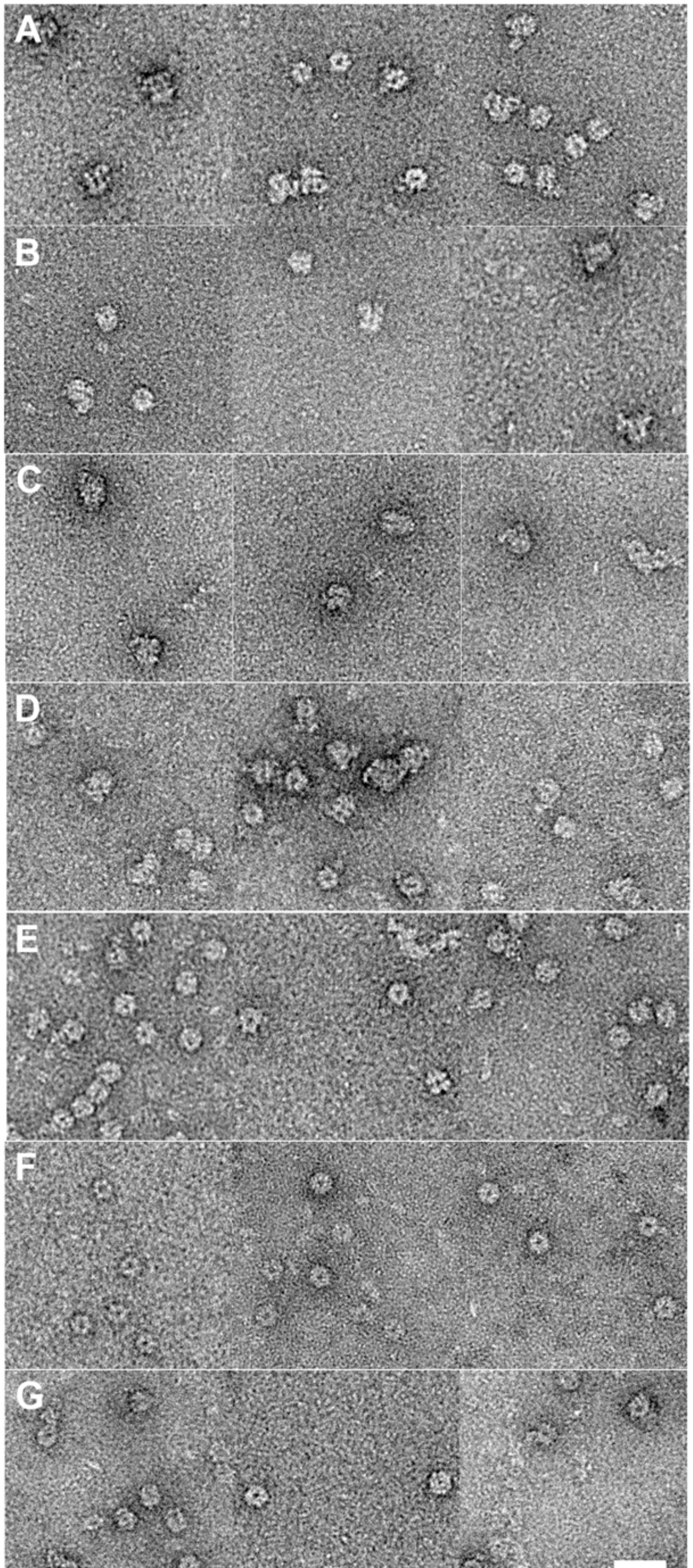

Supplement: Supplementary file 1 [file pbio.0050093.sg001.pdf]
