# Supplementary material for: Elucidating the Ticking of an In Vitro Circadian Clockwork
Source: PLoS Biol. 2007 Mar 27;5(4):e93. doi: 10.1371/journal.pbio.0050093 (PMC1831719; doi:10.1371/journal.pbio.0050093)
Supplement: Supplementary file 3 [file pbio.0050093.sg003.pdf]

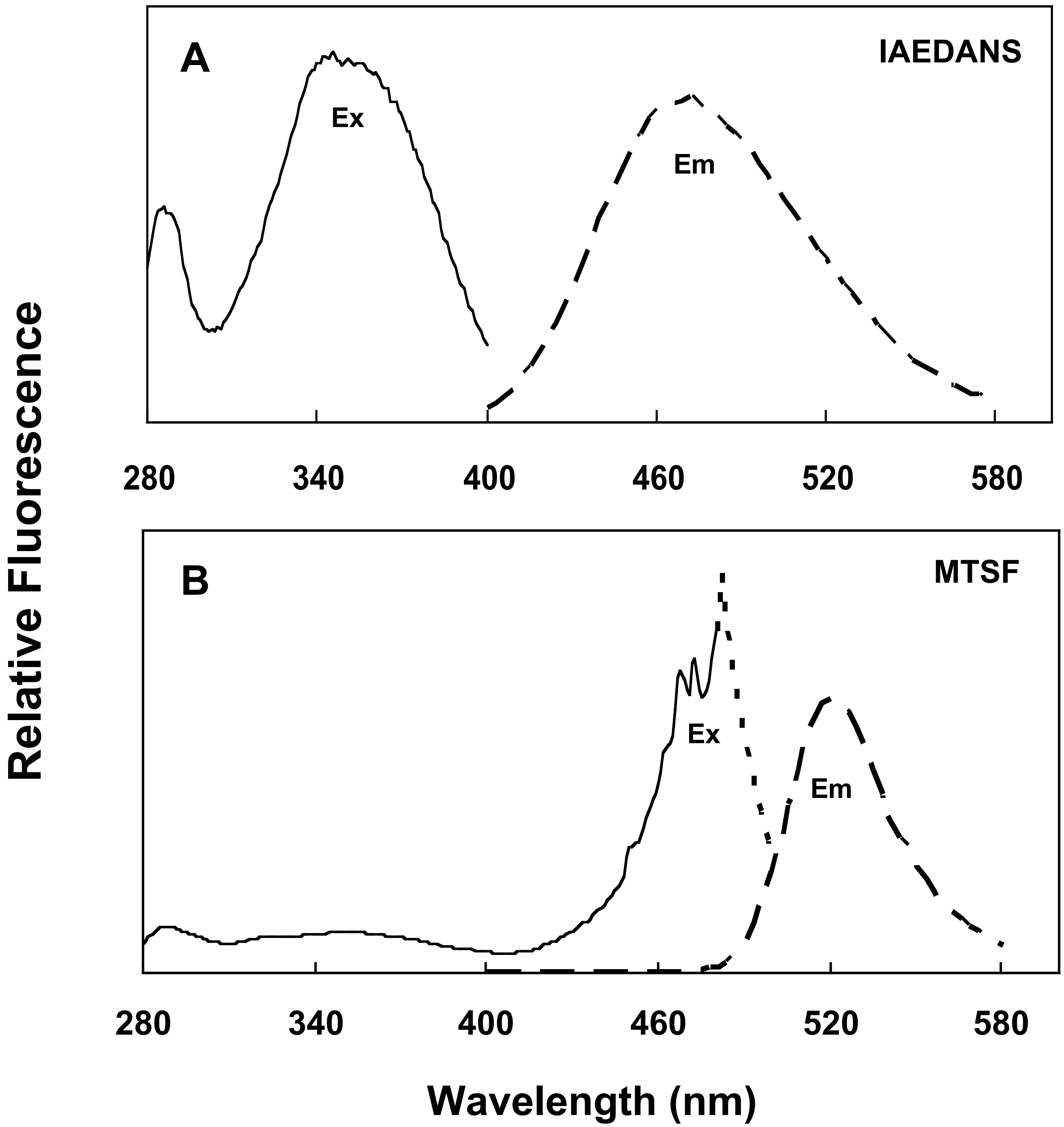

Figure 3 for Supplementary Information Section
