# Supplementary material for: Elucidating the Ticking of an In Vitro Circadian Clockwork
Source: PLoS Biol. 2007 Mar 27;5(4):e93. doi: 10.1371/journal.pbio.0050093 (PMC1831719; doi:10.1371/journal.pbio.0050093)
Supplement: Supplementary file 4 [file pbio.0050093.sd001.doc]

**Supporting Information for " Elucidating the Ticking of an *in vitro* Circadian Clockwork"**

Tetsuya Mori*, Dewight R. Williams*, Mark O. Byrne*, Ximing Qin, Martin Egli, Hassane S. Mchaourab, Phoebe L. Stewart, and Carl Hirschie Johnson

*These authors contributed equally

**Model Description**

The model consists of KaiC hexamers with a specified maximum number of phosphates per monomer (e.g., 2 or 3). Hexamerization in the presence of ATP is assumed to be a rapid process relative to the oscillation timescale and is assumed not to participate in the oscillatory dynamics. Therefore, we neglect explicit hexamer formation and dissociation in the model, although we allow for the possibility of “monomer exchange” among hexamers. Monomer exchange may be associated with probabilistic monomer dissociation/association rates from the hexamer (in the presence of ATP) resulting in a steady-state monomer pool whose relative fraction is set by the relative on and off rates. For simplicity we do not assume an explicit mechanism for the monomer dynamics and allow monomers from different hexamers to “exchange” at some unknown timescale that is a free parameter in the model. We explicitly track each monomer on each hexamer and its degree of phosphorylation (0 to k).

We assume that KaiA stochastically binds and unbinds rapidly from KaiC hexamers. When KaiA is bound to KaiC, the monomers in the complex are phosphorylated at a faster rate than in the absence of KaiA, so that this protein acts as an amplifier of (auto) phosphorylation and may also decrease the rate of (auto) de-phosphorylation. We have allowed for the possibility of a phenomenological memory term of KaiA binding so that following unbinding of KaiA from KaiC, the hexamer remains in the enhanced phosphorylation state for some relatively small time period relative to the oscillation timescale. We assume that KaiB associates/disassociates with the KaiC hexamer when the total degree of phosphorylation of the hexamer exceeds some threshold (T) so that the interaction of KaiB and KaiC is contingent upon conformational changes resulting from hyper-phosphorylation. It has been shown that there is an asymmetric association of KaiB with phosphorylated KaiC versus non-phosphorylated KaiC [21].

We assume that binding of KaiB irreversibly alters the conformation of the KaiC hexamers (KaiC → KaiC*) instantaneously (on the oscillation timescale) so that KaiB operates as a sensor of hyper-phosphorylation. We assume that the rates of binding/unbinding of KaiB to hyper-phosphorylated KaiC are also rapid (minute timescales for rise times [21]). The altered KaiC* hexamers may also form complexes with KaiA (and/or KaiB). We assume that the KaiA-KaiC* (and KaiC*) complexes are essentially non-phosphorylatable or have a slower rate of phosphorylation than (auto) de-phosphorylation so that the amplification of hexamer phosphorylation by KaiA is no longer applicable.

Based on the recent suggestion of monomer exchange among KaiC hexamers [21], the model allows for different rates of monomer exchange between KaiC-KaiC, KaiC-KaiC*, and KaiC*-KaiC* hexamers (Fig. 3, double-headed arrows), i.e., between all the possible KaiC states. These states are simplified in Figure 3 as “α, β, χ, and δ,” but in reality, there can be as many as 24,575 distinct KaiC hexamers at a given time (k=3). Assuming that a maximum of k phosphates can be bound per monomer, there are k + 1 phosphorylation states for each monomer (0,1,2,...,k). If each monomer is independent, there are (k+1)^6^ states for any hexamer (729 for k = 2). In addition these can be bound to KaiA, KaiB, or both KaiA and KaiB, giving 3*(k+1)^6^ states. We assume two conformations so there are actually 6*(k+1)^6^ states in this model (*4374 for k = 2; 24,576 states for k =3*). The distinct physical states are less than this if there is rotational symmetry of the KaiC hexamer, since multiple states with the same total number of phosphates are physically equivalent with this additional assumption. At any given time the hexamer population will be distributed in these states and the statistical distribution of phosphorylation from all monomers will be oscillating with a circadian period of ~24 hours. The rates of exchange could also be phosphorylation dependent such that hyper-phosphorylated KaiC (KaiC*) has a slower exchange rate than non-phosphorylated KaiC (KaiC*). Finally non-phosphorylated KaiC* relaxes to non-phosphorylated KaiC at some unknown rate.

**Reactions**

**1. Hexameric reactions** (n labels the number of phosphates on the hexamer, M_max_ is the maximum number of phosphates per monomer, T is the threshold total phosphorylation of the hexamer for KaiB binding):

KaiA binding and unbinding to KaiC hexamer (with or without KaiB bound):

(1) C_(n)_ + A ↔ A C_(n)_	(n = 0, 6 × M_max_)

KaiB binding and unbinding to KaiC hexamer (with or without KaiA bound) above threshold phosphorylation level T, followed by an irreversible change in conformational state.

(2) C_(n)_ + B ↔ B C_(n)_ → B C*_(n)_ ; (n > T)

KaiB binding and unbinding to KaiC* (with or without KaiA bound)

(3) C*_(n)_ + B ↔ B C*_(n)_ (n > T)

KaiA binding and unbinding to KaiC* (with or without KaiB bound)

(4) C*_(n)_ + A ↔ A C*_(n)_	(n = 0, 6 × M_max_)

KaiC* reverting to KaiC (with or without KaiA bound)

(5) C*_(0)_ → C_(0)_

**2. Monomeric reactions** (k refers to the number of phosphates on the monomer M):

Phosphorylation:

(1) M _(k)_ → M _(k + 1)_	(k = 0, M_max_ -1), rate is hexamer-state dependent

De-Phosphorylation:

(2) M _(k)_ → M _(k - 1)_	(k = 1, M_max_), rate is hexamer-state dependent

Hexamer-dependent phosphorylation rates (R _p_) of monomers:

R _p_^1^ = free KaiC

R _p_^2^ = KaiA-KaiC (“fast”, R_p_2 >> R_p_1)

R* _p_^1^ = free KaiC* , KaiA-KaiC*, KaiB-KaiC*, KaiA-KaiB-KaiC* (“slow”, R_p_*1 << R_p_1)

Hexamer-dependent de-phosphorylation (R _-p_) rates:

R _–p_^1^ = free KaiC , KaiA-KaiC	(“slow”, R_-p_1 << R_p_2 )

R* _–p_^1^ = free KaiC* = KaiA-KaiC* = KaiB-KaiC* = KaiA-KaiB-KaiC* (“fast”, R_-p_*1 >> R*_p_1)

Monomer exchange across hexamers (M1 and M2 refer to arbitrary monomers on any hexamers, M_max_ is the maximum number of phosphates per monomer):

Exchange of monomers between KaiC and KaiC hexamers (free and bound)

(3) M1 _(k1)_ ↔ M2 _(k2)_ (k1, k2 = 0, M_max_)

Exchange of monomers between KaiC* and KaiC* hexamers (free and bound)

(4) M1*_(k1)_ ↔ M2*_(k2)_ (k1, k2 = 0, M_max_)

Exchange of monomers between KaiC and KaiC* hexamers (free and bound)

(5) M1*_(k1)_ ↔ M2_(k2)_	(k1, k2 = 0, M_max_)

Rates of monomer exchange may be KaiC (KaiC*) state dependent (e.g., bound or unbound to KaiA/KaiB, etc.).

**Implementation of the stochastic model**

Each hexamer and monomer is treated equivalently and independently. During each simulation step, each hexamer may undergo “hexameric” reactions (R_k_^H^ and k = 1,..h_max_) where R refers to a reaction, H refers to a hexamer reaction, k labels a specific reaction, and h_max_ is a label for the total number of hexamer reactions. Thus R_1_^H^ and R_2_^H^ may refer to the formation and dissociation of KaiC hexamers with KaiA, respectively. Similarly, each monomer may (independently) undergo a “monomeric” reaction (R_k_^M^ and k = 1,..,m_max_) so that R_1_^M^ and R_2_^M^ refer to monomer phosphorylation and de-phosphorylation reactions respectively. In a time interval δt, a hexamer may react in any allowable "hexameric reaction" (R_k_^H^) with respective probabilities per unit time P_k_^H^ such that Σ P_k_^H^ < 1 and (Σ P_k_^H^ – 1) is the probability that no allowable hexameric reaction occurs. Also in any time interval δt the 6 monomers of the hexamer are each (independently) permitted to undergo a monomer reaction (R_k_^M^) with associated probability per unit time P_k_^M^, the value of which depends on the conformation and binding status of the hexamer of which it is a constituent. The probabilities are expressed per unit time per molecule. In a time δt, each hexamer and each monomer is permitted to undergo only a single reaction (or does not participate in any reaction, depending on P_k_ and the computer-generated random number(s) selected on [0,1]). Starting with an initial number of molecules K(t), the expected number of molecules that have undergone an allowable reaction R_k_ in a small interval of time δt is K(t)*P_k_*δt, such that 0 < P_k_*δt < 1 << K. In the formulation given here we keep track of the state of each individual hexamer. The resulting (time-dependent) behavior of is equivalent to the usual stochastic formulation of the relevant differential equations since the fundamental stochastic hypothesis is employed [41]. While the reaction probability density is usually derived, here we explicitly numerically simulate, for each timestep, the probability that a reaction occurs for a given molecule and the probability that the reaction is of a specific type.

To account for potential exchange of monomers between different hexamers we define a monomer exchange probability per unit time (P_ex_^M^) that depends on the conformational state of both KaiC hexamers that are participating. A monomer is selected from a hexamer for potential exchange. A random monomer from the remaining hexamers is selected and they are exchanged with probabilities per unit time depending on the conformational state of both hexamers. As initial conditions we choose probabilities per unit time selecting a timestep relevant to the fastest reaction and timescale suggested by experiments. The association/dissociation kinetics of KaiA are to be likely the fastest reactions in this system (minute timescales) and we selected various timesteps of order seconds to minutes so that initial conditions are reported as probabilities of reaction per second. The circadian period of ~24 hours is a consequence of the oscillatory behavior (for various relative rate constants) in the model and, in the absence of constraints on the rate parameters, we are free to generate such a period by an appropriate choice of model parameters.

**Matrix Formulation**

Hexamer kinetics are explicitly modeled using an N by (6 + 3) matrix, where N refers to the number of hexamers (rows of the matrix). The first six columns refer to the six monomers within the hexamer, and the matrix value is the number of phosphates on that monomer taking integer values in the range [0, k]. The final 3 columns label the association status of the hexamer and its conformation. For example the seventh column has an index identifying whether the hexamer is bound or unbound to KaiA (e.g., 0 or 1), the eighth whether bound or unbound to KaiB, and the ninth whether the conformation is C or C*. The dynamics of hexamers are simulated using Monte Carlo. A discrete timestep updates all N hexamers and 6N monomers according to the selected probabilities (per time). Monomers are phosphorylated/de-phosphorylated with some pre-selected probabilities (per unit time) depending on whether they are in the state C or C*. Likewise KaiA or KaiB binds and unbinds to a hexamer with pre-defined probabilities (neither are assumed to be limiting). For KaiB, binding only occurs if the total number of phosphates on a monomer is greater than some threshold value. Monomers are allowed to exchange with some probability depending on the conformational state of both hexamers selected. The stochastic model was implemented in both Matlab and Fortran G77 (GNU) and is available upon request (from M.B.). The parameters and rate constants used in the manuscript figures are below.

**Model parameters and rates**

| **Parameters** |  |
| --- | --- |
| Number of hexamers | 2000 |
|  |  |
| Maximum # of phosphates | 3 |
| per monomer |  |
|  |  |
| Threshold for KaiB binding | 17 |

|  |  | ***Probability per second (per molecule)*** | | | |
| --- | --- | --- | --- | --- | --- |
| ***Reaction*** | ***Hexamer State*** | ***Fig. 4*** | ***Fig. 6*** | ***Fig. 7C*** | ***Fig. 7C*** |
|  |  |  | ***(dashed)*** | ***(control)*** | ***(16C)*** |
| Phosphorylation | KaiC | 7.03E-06 | same | same | same |
|  | KaiA-KaiC | 1.69E-04 | same | same | same |
|  | KaiC*, KaiB-KaiC* | 1.41E-07 | same | same | same |
|  | KaiA-KaiC* | 1.41E-07 | same | same | same |
| De-phosphorylation | KaiC | 2.81E-05 | same | same | same |
|  | KaiA-KaiC | 1.41E-06 | same | same | same |
|  | KaiC*, KaiB-KaiC* | 7.03E-05 | same | same | same |
|  | KaiA-KaiC* | 7.03E-05 | same | same | same |
| KaiA interactions | KaiA-KaiC, KaiA-KaiC* on | 2.25E-03 | same | same | same |
|  | KaiA-KaiC, KaiA-KaiC* off | 3.75E-03 | same | same | 3.75E-05 |
| KaiB interactions | KaiB-KaiC, KaiB-KaiC* on | 4.69E-04 | same | same | same |
|  | KaiB-KaiC, KaiB-KaiC* off | 8.44E-05 | same | same | 8.44E-07 |
| Relaxation | KaiC* -> KaiC | 1.88E-04 | same | same | 1.88E-06 |
| Monomer exchange | KaiC-KaiC | 3.75E-03 | 0 | 3.75E-03 | 3.75E-03 |
|  | KaiC*-KaiC* | 4.22E-03 | 0 | 4.22E-03 | 4.22E-03 |
|  | KaiC-KaiC* | 0.00E+00 | same | same | same |

**Sensitivity of the oscillatory system**

The existence of sustained oscillations is relatively insensitive to the on/off rates of KaiA-KaiC and KaiB-KaiC, monomer exchange rates (these must be fast enough for synchronization but there are a broad range of rates consistent with oscillations), and the relaxation kinetics for KaiC* to KaiC conversion. There is, however, a sensitive dependence of the oscillations on the phosphorylation and de-phosphorylation rates (in states C and C*, respectively). The model indicates that if the rate of phosphorylation is not sufficiently high for KaiC (and KaiA-KaiC) then oscillations will not generally occur. In other words, if the auto-phosphorylation rate was sufficiently fast then the oscillator might function in the absence of KaiA (with only KaiC and KaiB). It appears to be most likely that, given the apparent low percent of KaiA-KaiC complexes at any given time (~10%), KaiA alters a hexamer into a state of enhanced phosphorylation (and perhaps suppressed de-phosphorylation) for a timescale corresponding to at least the KaiA re-association time. We have explicitly modeled the system with and without the KaiA-KaiC memory dependent term. For the manuscript figures we used a memory of KaiA association of one timestep (~ 3.5 minutes); the KaiC hexamer was phosphorylated and de-phosphorylated as if KaiA were still bound for this additional timestep following unbinding of KaiA. As might be expected this phenomenological term allows for robust oscillations with a small percent of KaiA bound to KaiC at any given time and with an auto de-phosphorylation rate exceeding that of auto-phosphorylation (with no KaiA bound).

**Perturbation Test of the Model by Modulating Kai Protein Concentrations**

Kageyama and coworkers examined the phosphorylation rhythm of KaiC under variation in the total concentration of KaiA, KaiB and KaiC simultaneously (using a fixed stoichiometry) and on varying KaiA and KaiB protein concentrations separately while holding the remaining protein concentrations fixed [21]. For the formulation described above, the initial number of KaiC molecules and volume were assumed to be fixed. The reaction parameters (probabilities per unit time) for bi-molecular interactions vary inversely as the volume. Explicitly, the reaction probability (*c_μ_*) for hard sphere bimolecular interactions in thermal equilibrium at temperature *T* is given by

$c_{\mu}=\frac{1}{V}{\pi d}^{2}\sqrt{\left( 8\text{kT}/{\pi m} \right)}\text{exp}(-u\text{*/}\text{kT})$

where *V* is the volume, *d* is a distance scale characterizing a minimum collision distance, *k* is Boltzmann’s constant, *m* is the reduced mass, and *u** is the effective activation energy for a reaction [42]. For simulating the effect of a total concentration change with a fixed number of molecules the association rates of the KaiA-KaiC, KaiB-KaiC and monomer exchange rates were altered by the appropriate volume scale factors assuming an initial set of “control” values (e.g., the Figure 4 manuscript rate parameters). Since these “control” rates are not precisely known, the simulated perturbation behavior is representative of a qualitative effect of concentration on the rhythm of KaiC phosphorylation and is not intended to directly quantitatively match the results presented in [21].

The experimental concentration perturbation behavior of [21] indicates that the oscillatory dynamics (period, amplitude and phase) of KaiC phosphorylation are unaffected by increased concentrations and are significantly effected by decreased concentrations, of both total and relative protein levels. In both the relative and total perturbation experiments, decreasing concentrations result in progressively longer periods of oscillations, damping, and subsequent loss of oscillations. The reduction of protein concentrations is interpreted in the model as a reduction in the effective association rates since these rates scale with the diffusive encounter probability. For example, the effect of reduced KaiA-KaiC binding rates from decreasing concentrations of KaiA while keeping KaiB and KaiC fixed results (in simulations) in a longer period, loss of oscillations, and finally a steady-state level of phosphorylation set by the ratio of auto-phosphorylation to auto de-phosphorylation (Fig. S4A). In contrast, decreasing KaiB sufficiently while holding KaiA and KaiC fixed results in a high steady state phosphorylation level since KaiA is still amplifying the auto-phosphorylation rate of KaiC (Fig. S4B). The total concentration perturbation (KaiA, KaiB and KaiC) behavior on the oscillator is then interpreted in the context of the model as the combined result of altering both the KaiA and KaiB encounter probabilities (and monomer exchange rates, assuming these also scale with diffusive encounter). As the total concentrations of KaiA, KaiB and KaiC are lowered (Fig. S4C) the effect of decreased KaiA association sets the overall qualitative characteristics of the oscillations. The expected effect of increased total concentration would be to increase the KaiA-KaiC and KaiB-KaiC association rates. In the simulations used for this set of control parameters, increasing the total concentration resulted in faster oscillations due to increased KaiA association and its effect on phosphorylation. However, if a longer KaiA memory term is employed an increased association will have no effect on the oscillations and the global concentration increases will show no obvious change in the KaiC phosphorylation rhythm. To quantitatively match the experimental results of [21] will require characterizing the phosphorylation dynamics and concentration perturbation behavior over the range of all potential physiological rates (e.g., by random sampling of rate parameters). It should be noted that initial conditions in [21] are approximately 40-60% phosphorylated KaiC whereas in these simulations all the hexamers are initially completely un-phosphorylated.
